# miR-630 targets IGF1R to regulate response to HER-targeting drugs and overall cancer cell progression in HER2 over-expressing breast cancer

**DOI:** 10.1186/1476-4598-13-71

**Published:** 2014-03-24

**Authors:** Claire Corcoran, Sweta Rani, Susan Breslin, Martina Gogarty, Irene M Ghobrial, John Crown, Lorraine O’Driscoll

**Affiliations:** 1 School of Pharmacy and Pharmaceutical Sciences & Trinity Biomedical Sciences Institute, Trinity College Dublin, Dublin 2, Ireland; 2 Department of Medical Oncology, Dana-Farber Cancer Institute, Harvard Medical School, Boston, MA, USA; 3 Department of Oncology, St. Vincent’s University Hospital, Dublin 4, Ireland

**Keywords:** MicroRNA-630, IGF1R, Biomarker, Breast cancer, Drug resistance, Cancer cell aggression

## Abstract

**Background:**

While the treatment of HER2 over-expressing breast cancer with recent HER-targeted drugs has been highly effective for some patients, primary (also known as innate) or acquired resistance limits the success of these drugs. microRNAs have potential as diagnostic, prognostic and predictive biomarkers, as well as replacement therapies. Here we investigated the role of microRNA-630 (miR-630) in breast cancer progression and as a predictive biomarker for response to HER-targeting drugs, ultimately yielding potential as a therapeutic approach to add value to these drugs.

**Methods:**

We investigated the levels of intra- and extracellular miR-630 in cells and conditioned media from breast cancer cell lines with either innate- or acquired- resistance to HER-targeting lapatinib and neratinib, compared to their corresponding drug sensitive cell lines, using qPCR. To support the role of miR-630 in breast cancer, we examined the clinical relevance of this miRNA in breast cancer tumours versus matched peritumours. Transfection of miR-630 mimics and inhibitors was used to manipulate the expression of miR-630 to assess effects on response to HER-targeting drugs (lapatinib, neratinib and afatinib). Other phenotypic changes associated with cellular aggressiveness were evaluated by motility, invasion and *anoikis* assays. TargetScan prediction software, qPCR, immunoblotting and ELISAs, were used to assess miR-630’s regulation of mRNA, proteins and their phosphorylated forms.

**Results:**

We established that introducing miR-630 into cells with innate- or acquired- resistance to HER-drugs significantly restored the efficacy of lapatinib, neratinib and afatinib; through a mechanism which we have determined to, at least partly, involve miR-630’s regulation of IGF1R. Conversely, we demonstrated that blocking miR-630 induced resistance/insensitivity to these drugs. Cellular motility, invasion, and *anoikis* were also observed as significantly altered by miR-630 manipulation, whereby introducing miR-630 into cells reduced cellular aggression while inhibition of miR-630 induced a more aggressive cellular phenotype.

**Conclusions:**

Taken together, our findings suggest miR-630 as a key regulator of cancer cell progression in HER2 over-expressing breast cancer, through targeting of IGF1R. This study supports miR-630 as a diagnostic and a predictive biomarker for response to HER-targeted drugs and indicates that the therapeutic addition of miR-630 may enhance and improve patients’ response to HER-targeting drugs.

## Background

Approximately a quarter of all breast cancers are associated with the HER2-overexpression [1]. Amplification of the HER2 gene has been associated with more aggressive disease and, prior to the development of HER-targeted therapies, had an overall poor prognosis [2]. Since the initial development of trastuzumab (Herceptin); the use of lapatinib (Tykerb) a small-molecule tyrosine kinase inhibitor (TKI) that dually targets human epidermal growth factor receptor 2 (HER2) and epidermal growth factor receptor (EGFR/HER1)) have vastly improved clinical benefit in recent years [3,4]. Unfortunately, evidence of innate- or acquired- resistance to these drugs [5-8] indicates that, despite their initial success in the treatment of HER2-overexpressing cancers, their use in the clinic is becoming somewhat compromised. Newer HER-targeting drugs including neratinib (HK1-272) and afatinib (BIBW 2992), both of which are irreversible oral small molecule TKIs of target HER2, EGFR/HER1 and HER4, are producing promising results in clinical trials [9,10]. Unfortunately, it is probable that these drugs will eventually be faced with similar problems due to resistance as that of their predecessors.

There is an urgent need to identify predictive biomarkers for HER-targeting drugs in order to improve patients’ stratification and, subsequently, patients’ outcome. microRNAs (miRNAs) are small (approximately 18–25 nucleotides long) non-protein coding RNAs that have been associated with regulating gene transcription at a post-translational level [11]. In general, miRNAs are considered as negative regulators of gene expression. For example, miRNAs acting as tumour suppressors are responsible for controlling the levels of genes promoting tumourigenesis and are often decreased in cancerous compared to normal cells [12].

miR-630 has previously been reported to regulate cisplatin-induced cell death in both non-small cell lung cancer and head and neck cancer [13,14]. In a separate study, inhibition of miR-630 in lung cancer cells was found to be associated with an induction of cellular migration and invasion [15]. Furthermore, the over expression of miR-630 has recently been implicated in degradation of Insulin Growth Factor Receptor 1 (IGF1R) mRNA and protein levels and subsequent enhanced apoptosis in pancreatic cancer cells [16].

In this study, we aimed to investigate the relevance of miR-630 in breast cancer and its potential to regulate response to HER-targeting agents and cancer cell aggression. Through initial analysis of our innately and acquired drug-resistant cell lines we identified miR-630 as being significantly decreased compared to drug-sensitive age-matched parent cells. The relevance of further studying this target was confirmed by the finding that miR-630 was significantly decreased in breast cancer tissue compared to matched peritumour tissue. Our subsequent studies suggest that manipulation of miR-630 can influence cell response to HER-targeted agents, lapatinib, neratinib and afatinib apparently via its regulation of IGF1R. Motility, invasion and *anoikis* assays indicate that miR-630 may also play part in regulating the metastatic phenotype of HER2 over-expressing breast cancer cells.

## Methods

### Cell culture and treatments

SKBR3 and HCC1954 cells, obtained from ATCC, were cultured in RPMI-1640 (Sigma-Aldrich) with 10% FCS (PAA) and 1% L-glutamine (Sigma-Aldrich). MDA-MB-453 were cultured in McCoys 5A with 10% FCS and 1% L-glutamine (Sigma-Aldrich). Lapatinib-resistant SKBR3 and HCC1954 cells (SKBR3-LR and HCC1954-LR, respectively) were established by continuously exposing cells to lapatinib, starting with 5 nM and increased stepwise to 250 nM over 6 months. Similarly, neratinib-resistant cells (HCC1954-NR) were established by continuously exposing cells to neratinib, increasing stepwise to 250 nM for over 4 months. Age-matched parent cells (SKBR3-Ag, HCC1954-Ag) were maintained in culture, in parallel, but were not exposed to drug. Lapatinib, neratinib and afatinib were obtained from Sequoia Research Chemicals Ltd. (Pangbourne UK).

### RNA isolation from conditioned medium

For analysis of extracellular miR-630 levels, conditioned medium (CM) was collected, centrifuged and filtered, as we have previously described [17].

### miR-630 analysis in cells & conditioned medium

Total RNA was isolated from cell lines and CM using TriReagent (Sigma-Aldrich). cDNA was prepared from 10 ng cell-derived and 4 μl CM-derived total RNA, respectively, as we described previously [18]. miR-630 (001563, ABI, UK) was quantified using the cycle threshold (C_T_) adjusting to the levels of U6 snRNA (001973, ABI, UK) used as an endogenous control.

### Assessment of miR-630 expression in patient derived tumour tissue

miR-630 expression in breast cancer [all breast tissue (n *=* 56) and HER2+ breast tissue (n *=* 6)] was determined based on previous miRNA profiling of breast tumours and matched peritumours using a publically available data set (GSE40525) on Gene Expression Omnibus (GEO) (http://www.ncbi.nlm.nih.gov/geo/) [19]. microRNA expression levels, p-values and Log fold change (Log FC) between experimental conditions were determined using the GEO2R analysis function.

### microRNA inhibition/mimic manipulation in cells

SKBR3-Ag and HCC1954-Ag cells were transfected with miR-630 inhibitor (Cat #4464084, ID: MH11552, ABI, UK) or miRNA inhibitor negative control (Cat #4464076). These were used at a final concentration of 30 nM and transfected using lipofectamine 2000 (Invitrogen). 48-72 hrs post-transfection cells harvested for RNA and protein and functional assays were performed. Similarly, SKBR3-LR, HCC1954-LR and MDA-MB-453 cells were transfected with miR-630 mimic (Cat #4464066, ID MH11552, ABI, UK) or miRNA mimic negative control (Cat #4464058).

### Assessing effects of miR-630-regulated cellular response to HER-targeting drugs

Following transfection with miR-630 inhibitor, miR-630 mimic or their relevant negative controls, cells were exposed to their approximate IC_50_ concentrations of lapatinib (as indicated Tables 1 and 2) previously been determined by ourselves and/or others [20-23]. Fixed concentrations of neratinib and afatinib were also used (see Tables 1 and 2). Following 72 hrs incubation with the given drug, cell proliferation was assessed using acid phosphatase analysis.

**Table 1:** Inhibition of miR-630 reduces sensitivity to HER-targeted drugs, lapatinib, neratinib and afatinib

**Drug**	**Drug Conc.**	**Cell line**	**% Cell -proliferation drug + NC Inhibitor**	**% Cell proliferation drug + miR-630 Inhibitor**	**% Resistance induced by miR-630 inhibitor**	**p-value**
Lapatinib	0.05 μM	SKBR3-Ag	50.0 ± 9.6	63.6 ± 9.1	***13.6 ± 0.8***	0.002
Lapatinib	0.7 μM	HCC1954-Ag	40.8 ± 3.6	63.7 ± 4.8	***22.9 ± 6.7***	0.039
Neratinib	0.005 μM	SKBR3-Ag	34.7 ± 6.4	43.2 ± 7.4	***8.6 ± 1.6***	0.017
Neratinib	0.005 μM	HCC1954-Ag	43.2 ± 2.3	58.2 ± 2.4	***15.1 ± 3.0***	0.019
Afatinib	0.005 μM	SKBR3-Ag	47.7 ± 5.2	62.3 ± 4.6	***14.6 ± 1.7***	0.002
Afatinib	0.005 μM	HCC1954-Ag	70.4 ± 8.3	82.1 ± 9.0	***11.7 ± 2.3***	0.019

**Table 2:** Over-expression of miR-630 restores sensitivity to lapatinib, neratinib and afatinib in cells with either acquired- or innate resistance to HER-targeting drugs

**Drug**	**Drug Conc.**	**Cell line**	**Drug + NC mimic**	**Drug + miR-630 mimic**	**% Anti-proliferative benefit with miR-630 mimic**	**p-value**
Lapatinib	3 μM	SKBR3-LR	82.5 ± 1.4	69.5 ± 1.2	***12.9 ± 1.4***	0.011
Lapatinib	5 μM	HCC1954-LR	27.6 ± 6.3	17.8 ± 4.5	***9.7 ± 2.3***	0.026
Lapatinib	6 μM	MDA-MB-453	19.0 ± 0.9	9.9 ± 0.9	***9.1 ± 1.6***	0.015
Neratinib	0.5 μM	SKBR3-LR	63.3 ± 1.9	56.3 ± 1.2	***7.0 ± 1.0***	0.019
Neratinib	0.5 μM	HCC1954-LR	26.3 ± 4.6	22.3 ± 4.7	***3.9 ± 0.1***	0.000
Neratinib	0.5 μM	MDA-MB-453	45.3 ± 7.5	28.3 ± 3.0	***17.0 ± 4.9***	0.037
Afatinib	0.5 μM	SKBR3-LR	78.4 ± 1.5	70.4 ± 0.7	***8.4 ± 1.4***	0.031
Afatinib	0.5 μM	HCC1954-LR	30.5 ± 7.0	20.7 ± 6.5	***9.8 ± 0.5***	0.001
Afatinib	0.5 μM	MDA-MB-453	50.6 ± 8.3	31.4 ± 10.4	***19.2 ± 2.3***	0.007

### qPCR for IGF1R

Total RNA was isolated from cell lines using TriReagent (Sigma-Aldrich). cDNA was prepared from 1 μg total RNA. IGF1R (Hs99999020_m1, ABI, UK) was quantified using the threshold cycle (C_T_) adjusting to the levels of β-actin (4352933E, ABI, UK), established as not differing significantly in expression levels between cell populations being assessed and so suitable as endogenous control.

### Immunoblotting

Total protein (30-100 μg) were resolved on 7.5% SDS-PAGE and transferred to PVDF membranes (Bio-Rad Laboratories). The following primary antibodies were used: HER2 (Calbiochem); EGFR, IGF1Rβ (Cell Signalling Technology); β-actin (Sigma-Aldrich). Following incubation in the appropriate horseradish peroxidase-conjugated secondary antibodies (Cell Signalling Technology), the immunoblots were developed using chemiluminescence (Thermo Fisher) and detected on a Chemidoc exposure system (Bio-Rad Laboratories). Densitometry was performed on the bands of protein expression using NIH ImageJ software and normalised to the loading control (β-actin).

### Enzyme-linked immunosorbent assays (ELISAs)

ELISAs (R&D Systems) for p-IGF1R, p-HER2 and p-EGFR were used according to the manufacturer’s instructions. As recommended, concentrations of total protein used for the ELISAs were prepared as 700 μg/ml (for p-IGFR) and 20 μg/ml (for p-HER2 and p-EGFR), with 100 μl of each loaded per ELISA well. Thus, 70 μg/well of total protein was loaded for the p-IGFR ELISA and 2 μg/well of total protein was loaded for the p-HER2 and p-EGFR ELISAs.

### Cell motility –assessed using wound healing assays

Transfected cells (3 × 10^5^ cells/well for SKBR3 cell line variants and 2 ×10^5^/well for HCC1954 cell line variants) were seeded on 24-well plates and cultured for 24 hrs to confluency. Monolayers were scratched with a pipette tip and the resulting wounded areas were monitored as previously described [24]. Throughout the duration of monitoring wound closure, cells were maintained in low serum (1%) containing medium to reduce any influences due to proliferation.

### Cell migration and invasion –assessed using transwell inserts

Migration and invasion assays were performed and quantified as previously described [24]. Transfected cells (HCC1954 variants, 5×10^4^/insert; SKBR3-Ag, 1×10^6^/insert; SKBR3-LR, 5×10^5^/insert) were seeded in the upper compartment and allowed to migrate for 48 hrs (HCC1954 variants) and 72 hrs (SKBR3 variants), respectively. Cells were seeded in low serum (1%) containing medium in the upper chamber and 10% serum-containing medium in the well below the insert.

### Anoikis assay

Transfected cells (1×10^4^ cells/well) were seeded onto a 24-well plates coated with Poly(hydroxyethyl methacrylic) acid (Sigma-Aldrich) or 95% ethanol and were cultured for 48 hrs. Alamar blue dye (100 μl; Serotec, UK) was added/well and absorbance read at 570 nm; reference wavelength, 600 nm.

### Statistical and bioinformatics analysis

Online miRNA target prediction software (TargetScan Human Release 6.2) was used to identify proteins potentially regulated by miR-630. Statistical analysis was performed in Excel. P-values were generated using Student’s T-tests, with p <0.05 considered as statistically significant. GraphPad Prism 5.0 was used for graph generation (Graph Pad Software Inc, La Jolla, USA).

## Results

### Changes in both intracellular and extracellular miR-630 levels are associated with acquired-resistance to HER-targeting drugs

**Figure 1:**
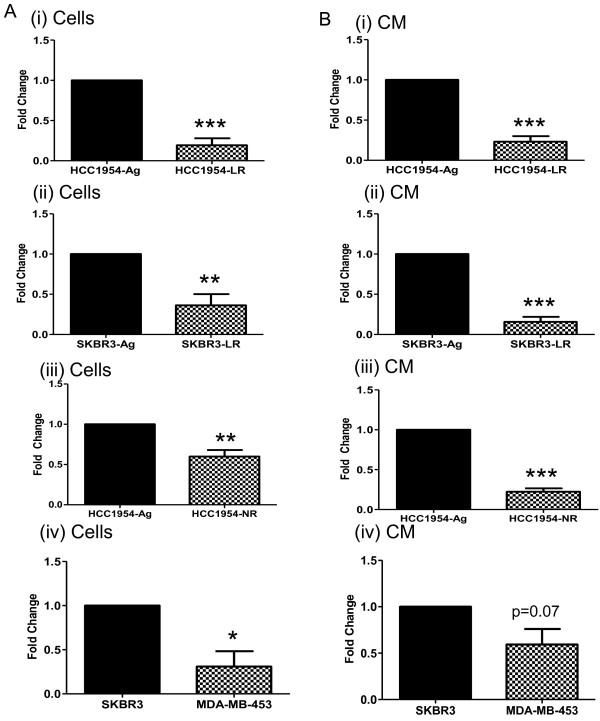
**miR-630 expression decreases with both acquired- and innate resistance. (A)** miR-630 expression was significantly decreased in cells with acquired lapatinib resistance (**(i)** HCC1954-LR and **(ii)** SKBR3-LR) and acquired neratinib-resistance (**(iii)** HCC1954-NR) compared to their age-matched control cells (HCC1954-Ag, SKBR3-Ag). miR-630 expression was also significantly decreased in cells with innate resistance to lapatinib (**(iv)** MDA-MB-453) compared to cells with innate sensitivity (SKBR3). **(B)** Extracellular expression of miR-630 was also assessed in the corresponding conditioned medium of these cells and trends followed that of the cells. All results represent biological repeats n = 3 ± SEM, where *p <0.05, **p <0.01, ***p <0.001.

miRNA profiling of parent (drug sensitive) and lapatinib-resistant cell lines, using TaqMan low density arrays, identified miR-630 as differentially expressed. The differential expression of miR-630 was validated using qPCR, whereby miR-630 was found to be significantly decreased in HCC1954-LR (Figure 1A (i); p <0.001) and SKBR3-LR (Figure 1A (ii); p <0.01) cells compared to their corresponding age-matched parent cells. Similarly, miR-630 expression was significantly decreased in neratinib-resistant HCC1954-NR (Figure 1A (iii); p <0.01). There was also a slight, but unsubstantial (1.25 fold) decrease of miR-630 in trastuzumab-resistant SKBR3 cells (*not shown*). To investigate whether miR-630 may be associated with innate resistance, in addition to acquired resistance, we next determined its levels in innately-resistant MDA-MB-453 cells [22]. Here a significant decrease in miR-630 was demonstrated when compared to innately-sensitive SKBR3 cells (Figure 1A (iv), p <0.05). Interestingly, the extracellular levels of miR-630 in the corresponding CM also reflected that of the acquired-resistant cells i.e. HCC1954-LR, SKBR3-LR and HCC1954-NR (Figure 1B (i-iii), p <0.001), with a trend towards a decrease in MDA-MB-453 compared to SKBR3 CM (Figure 1B (iv), p =0.07).

### miR-630 is decreased in tumour compared to peritumour breast cancer tissues

**Figure 2:**
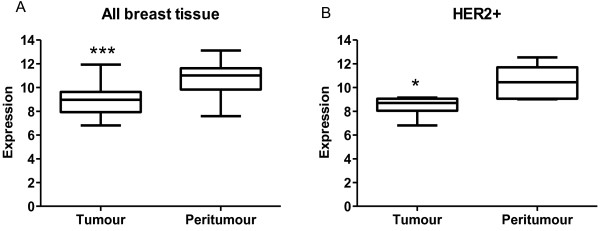
**Clinical relevance of miR-630 in breast cancer.** Using a publically available data set (GSE40525) from Gene Expression Omnibus (GEO) the expression of miR-630 was determined in breast cancer tumour tissues compared to matched peritumour tissue. **(A)** miR-630 was significantly decreased in all breast tumours (n = 56) and also in **(B)** HER2+ breast cancer tissue (n = 6) compared to matched peritumours. *p <0.05, ***p <0.001

In order to assess the possible clinical importance of miR-630 and so relevance to further study this microRNA, using a publically available dataset (GSE40525) we investigated the expression of miR-630 in breast cancer tumour tissues versus matched peritumour tissues. A significant decrease in miR-630 expression in all breast cancer tumours compared to matched peritumours was observed (Log FC: 1.83; p <0.001) (Figure 2A). Subsequent analysis of those breast tissues that were HER2+ also displayed a significant decrease in miR-630 in HER2+ tumours compared to matched peritumour tissue (Log FC: 2.02, p <0.05) (Figure 2B).

### miR-630 regulates cellular response to HER-targeted drugs

miR-630 inhibition (when compared to the negative control inhibitor) caused significant increase in resistance/insensitivity to the anti-proliferative affects of each of lapatinib, neratinib and afatinib (Table 1). To ensure that an unbiased functional assessment of miR-630 was performed, we also transfected acquired-resistant cell lines (SKBR3-LR and HCC1954-LR) with miR-630 mimic. In terms of cellular response to the HER-targeted drugs assessed, miR-630 mimic was found to further enhance the anti- proliferative effects of all drugs assessed in HCC1954-LR and SKBR3-LR as well as innately-resistant MDA-MB-453 (Table 2).

### Phenotypic changes in HER2-overexpressing cells including motility, invasion and resistance to anoikis are also regulated, at least in part, by miR-630

**Figure 3:**
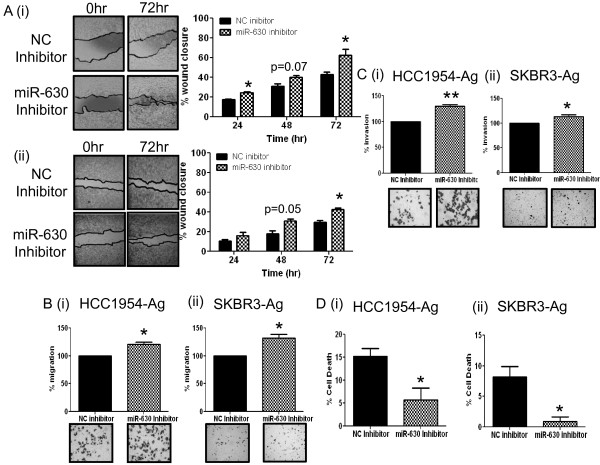
**Inhibition of miR-630 increased cell motility, migration, invasion and resistance to *anoikis.*** Following transfection with miR-630 inhibitor or a negative control (NC) inhibitor in **(i)** HCC1954-Ag and **(ii)** SKBR3-Ag **(A)** motility, assessed by wound-healing assay **(B)** migration, through transwells; **(C)** invasion, through ECM-coated transwells; and **(D)***anoikis*, on poly(hydroxyethyl methacrylic) acid coated plates; were found to be significantly changed as a consequence of miR-630 inhibition. Results represent n = 3 ± SEM, where *p <0.05, **p <0.01.

**Figure 4:**
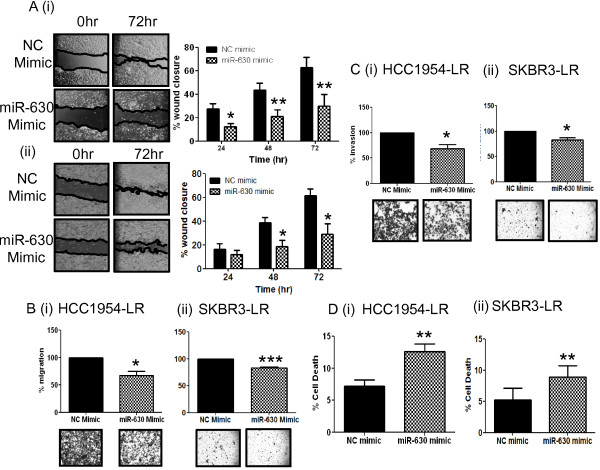
**Over-expression of miR-630 in lapatinib resistant cells decreases cell motility, migration, invasion and *anoikis.*** Following transfection with miR-630 mimic or a negative control (NC) mimic in HCC1954-LR **(i)** and SKBR3-LR **(ii) (A)** motility, assessed by wound-healing assay; **(B)** migration, through transwells; **(C)** invasion, through ECM-coated transwells; **(D)***anoikis*, on Poly(hydroxyethyl methacrylic) acid coated plates; were found to be significantly altered as a consequence of miR-630 induced expression. Results represent n = 3 ± SEM, where *p <0.05, **p <0.01, ***p<0.001.

We also observed that inhibition of miR-630 in HCC1954-Ag and SKBR3-Ag, compared to their respective negative controls, was associated with increased motility as evaluated via wound-healing assays (Figure 3A (i) p <0.05 & (ii) p <0.05 - after 72 hrs); increased migration through transwells (Figure 3B (i) p <0.05 & (ii) p <0.05); increased invasion through extracellular matrix-coated transwells (Figure 3C (i) p <0.01 & (ii) p <0.05); and resistance to *anoikis* (Figure 3D (i) p <0.05 & (ii) p <0.05). Conversely, miR-630 mimic transfection in HCC1954-LR and SKBR3-LR cells was associated with opposite effects i.e. decreased cellular motility (Figure 4A (ii) p <0.001 and (ii) p <0.05); decreased migration (Figure 4B (i) p <0.05 and (ii) p <0.01); decreased invasion (Figure 4C (i) p <0.05 & (ii) p <0.05); and increased sensitivity to cell death by *anoikis* (Figure 4D (i) p <0.01 and (ii) p <0.01).

### Proposed mechanism of action

**Figure 5:**
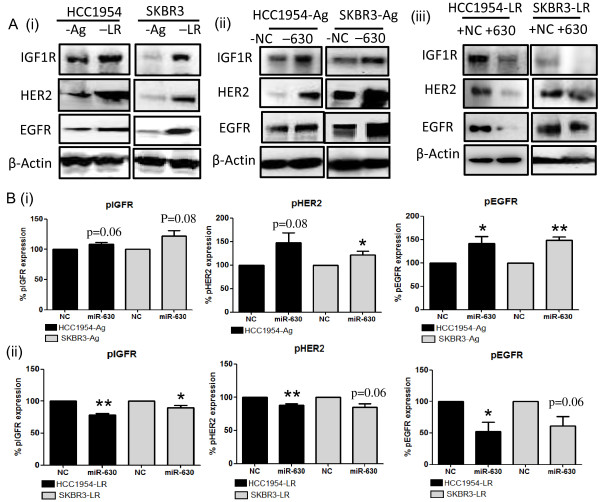
**miR-630’s mechanism of action.** IGF1R was predicted as a direct target of miR-630. **(A) (i)** Initially the expression of IGF1R, HER2 and EGFR was assessed in our acquired lapatinib-resistant cells (HCC1954-LR and SKBR3-LR) and was found to be increased compared to levels in their age-matched parent cells. **(ii)** Inhibition of miR-630 induced an increase in IGF1R, HER2 and EGFR expression in both HCC1954-Ag and SKBR3-Ag cells compared to levels in the negative control transfected cells. **(iii)** Conversely, induced expression of miR-630 in lapatinib-resistant HCC1954-LR and SKBR3-LR cells caused a decrease in IGF1R, HER2 and EGFR levels. **(B)** The phosphorylated forms of IGF1R, HER2 and EGFR were assessed using ELISAs, indicating that **(i)** inhibition of miR-630 induced an increase in pIGF1R, pHER2 and pEGFR, while **(ii)** over-expression of miR-630 decreased the pIGF1R, pHER2 and pEGFR levels. Representative immunoblots of n = 3 biological repeats. n = 3 ± SEM, where *p <0.05, **p <0.01, ***p < 0.001.

Through the use of target prediction software (TargetScanHuman Release 6.2), IGF1R was predicted to be regulated by miR-630. Initially we assessed the levels of IGF1R in our acquired lapatinib-resistant cells (HCC1954-LR and SKBR3-LR) and found a significant increase of this protein compared to the corresponding parent cells (Figure 5A (i)). We also observed that IGF1R to be increased in our neratinib-resistant cells (HCC1954-NR) compared to its corresponding parent cells *(not shown).*

Subsequently we demonstrate that when miR-630 is inhibited, and thus drug resistance and increased cell aggression is induced, expression levels of IGF1R protein are significantly increased in both HCC1954-Ag (p <0.05) and SKBR3-Ag (p <0.01) (Figure 5A (ii)). Similarly, conferred cellular sensitivity to the HER-targeting drugs and the reduction of cell aggression, that we observed by miR-630 mimic, was coupled with a significant reduction of IGF1R in HCC1954-LR (p <0.05) and SKBR3-LR (p <0.05) (Figure 5A (iii)).

To investigate if miR-630 acts by regulating mRNA transcription, the levels of IGF1R post-transfection with either the miR-630 inhibitor or miR-630 mimic were assessed by qPCR. Here we found that although a trend towards an increase in IGF1R mRNA existed following miR-630 inhibition compared to the negative control (Additional file 1: Figure S2), this did not reach statistical significance. Similarly, there was no significant decrease in IGF1R mRNA levels following miR-630 mimic transfection when compared to the levels in cells transfected with the negative control mimic (Additional file 1: Figure S2).

As HER2 and EGFR are the targets for all 3 drugs assessed, we also determined the protein levels of these specific targets using immunoblotting. Increased levels of HER2 and EGFR were observed in our acquired lapatinib-resistant cells (HCC1954-LR and SKBR3-LR) compared to the corresponding parent cells (Figure 5A (i)). Inhibition of miR-630 in HCC1954-Ag and SKBR3-Ag was associated with increased levels of HER2 and EGFR protein (Figure 5A (ii)). Conversely, miR-630 mimic transfection in HCC1954-LR and SKBR3-LR cells was associated with decreased levels of HER2 and EGFR expression (Figure 5A (iii)). All corresponding densitometry for immunoblots can be found in Additional file 2: Figure S1.

Next, we investigated if miR-630’s regulation of IGF1R and subsequent effects on HER2 and EGFR may be associated with altered phosphorylation states of some (or all) of these proteins. We observed that inhibition of miR-630 in SKBR3-Ag and HCC1954-Ag cells induced a significant increase (or in some cases tending towards significance) in pIGF1R, pHER2 and pEGFR (Figure 5B (i)). Conversely, miR-630 mimic transfection induced a decrease in phosphorylation of all three proteins assessed (Figure 5B (ii)).

## Discussion

Limited studies to date have reported on the role of miR-630 in cancer. Specifically, reports using the lung cancer cell line, A549, identified miR-630 as a regulator of cisplatin-induced cell death, with over-expression of miR-630 inducing chemoprotective properties [13]. Conversely, the authors of that study also identified that miR-630 failed to protect cells when treated with C_2_-CER, CdCl_2_, etoposide, mitoxantrone, and oxaliplatin and in some cases induced chemo-sensitivity [13]. In head and neck squamous cell carcinoma, also reporting on cisplatin-induced cell death, Huang et al. [14] reported that induced expression of miR-630 with cisplatin treatment of HNSCC cells dramatically decreased cell survival. Induced miR-630 expression decreased levels of anti-apoptotic genes, BCL2 and BCL2L2 whereas inhibition of miR-630 in HNSCC cells up-regulated these genes and was associated with increased cell survival [14]. The over expression of miR-630 in the pancreatic cancer cell line PANC-1 has recently been associated with decreased mRNA and protein levels of IGF1R as well as enhanced apoptosis [16] while the motility and invasion of the ANGPTL1 over-expressing lung cancer cell line (CL1-5/ANGPTL1) has been shown to be restored following miR-630 inhibition [15]. To the best of our knowledge, prior to our studies, miR-630 has never been associated with any subtype of breast cancer, with response/resistance to HER-targeted drugs and/or breast cancer cell aggression. Here we observed that miR-630 is associated with –and may be causally involved in– regulating resistance to HER-targeting drugs. Initially using our cell line models of acquired lapatinib-resistance (HCC1954-LR, SKBR3-LR) and neratinib-resistance (HCC1954-NR) and subsequently cells with innate resistance (MDA-MB-453), we observed a decrease in miR-630 expression.

The pursuit of extracellular predictive biomarkers offers potential for minimising invasive procedures such as tissue biopsies in the clinic. Previously, we have reported the existence of RNAs circulating extracellularly in serum/plasma from cancer patients [25]. We have also reported on the detection of circulating miRNAs [11,26,27]. In this study, we identified that medium conditioned by both lapatinib- and neratinib- resistant cell line variants of HCC1954 and SKBR3 cells (i.e. HCC1954-LR, SKBR3-LR, HCC1954-NR) demonstrated a significant decrease of miR-630 levels compared to conditioned medium from age-matched parent cells (HCC1954-Ag, SKBR3-Ag), correlating with the observation in the corresponding cell lines. Conditioned medium from MDA-MB-453 cells also showed a decrease to some extent in miR-630 levels compared to that in SKBR3 cells. This suggests relevance for miR-630 as an extracellular i.e. minimally-invasive predictive biomarker; a novel observation that warrants further investigation in future studies.

To support our in vitro findings, and so indicate the relevance of choosing miR-630 for further functional evaluation, our subsequent analysis was to determine the clinical relevance of miR-630. Evidently, miR-630 was decreased in breast cancer tumours compared to peritumour tissues which was also apparent when assessed in specifically HER2-overexpressing tumours.

Having established that reduced miR-630 expression levels are associated with both innate- and acquired- resistance, we progressed next to establishing if this was specific to lapatinib and neratinib alone or more generally associated with HER-targeting drugs. For this purpose, we also included afatinib in our analyses. Through inhibition of miR-630 (in sensitive parent cells) or its over-expression (in acquired lapatinib-resistant or innately-resistant cells) our studies identified a correlation between miR-630 expression and cellular response to all 3 HER-targeting drugs tested i.e. lapatinib, neratinib and afatinib.

To more comprehensively evaluate a functional relevance of miR-630 in HER2-overexpressing breast cancer we next investigated whether miR-630 may confer other phenotypic influences. Interestingly we observed that inhibition of miR-630, in sensitive parent SKBR3 and HCC1954 cells, was also associated with increased cell motility, migration, invasion and resistance to *anoikis*. On the contrary, miR-630 mimic transfection in acquired-resistant SKBR3-LR and HCC1954-LR cells resulted in a substantial block on these phenotypic changes. In agreement with our observations, increased cell migration and invasion have widely been reported to be associated with drug resistance [28-30]. Additionally, we have previously reported that drug resistance can also be coupled with increased resistance to *anoikis*[24]. Our findings in relation to miR-630’s involvement in regulating cell motility and invasion are supported by the recent study by Kou et al. [15] demonstrating that lung cancer cells (CL1-5) overexpressing ANGTL1 have reduced motile and invasive capabilities with increased levels of miR-630; whereas increased migration/invasion induced by shANGPTL1 in CL1-0 cells was associated with decreased miR-630 expression [15]. The authors of this study also demonstrated that inhibition of miR-630 in CL1-5/ANGTL1 cells restored invasion and migration [15]. Most breast cancers are of epithelial cells. Epithelial cells typically are attached to a basement membrane, rather than existing in suspension. For such cells to survive in suspension, as required for circulating tumour cells to be transported in the bloodstream or lymphatics and progress to forming tumour metastasis at secondary sites, these cells must evade a form of apoptosis termed *anoikis*[31]. In addition to invasion and migration, the ability of cells to resist *anoikis* and have anchorage-independent growth is a key contributing factor in cancer cell metastasis. Our observation that miR-630 may also regulate *anoikis*-resistance in breast cancer cells further indicates the potential importance of this miRNA in modulating overall breast cancer cell aggression.

Investigating the mechanism(s) by which miR-630 could be conferring these influences on sensitivity/resistance as well as altered cell aggression, IGF1R (which our bioinformatics analysis predicted as a target of miR-630) was found to be a directly influenced by miR-630 manipulation. Interestingly, elevated IGF1R expression, observed in our acquired lapatinib resistant cells (SKBR3-LR and HCC1954-LR) compared to their age-matched parent cells was inversely correlated with decreased miR-630 expression in the same resistant cells.

Advancing on this observation, we demonstrate that inhibition of miR-630 -resulting in increased resistance and metastatic phenotype in SKBR3 and HCC1954- was associated with increased IGF1R expression in both cell lines. Interestingly, in the presence of the miR-630 mimic, that induced an increase in sensitivity to HER-targeting drugs for acquired-resistant SKBR3-LR and HCC1954-LR cells as well as decreased cell aggressiveness, a reduction of IGF1R expression was observed. These findings correspond with that of several other reports that correlate IGF1R expression with resistance to HER-targeting drugs and other chemotherapy [32-36]. Furthermore, increased IGF1R has also been previously attributed with an increased aggressive phenotype [37-39]. Our studies, however, are the first in breast cancer to determine that the mechanism involved is, at least partly, regulated by aberrant miR-630 expression. This observation is supported by the recent study by Farhana et al. [16] who demonstrate that miR-630 pairs to a 7 nucleotide conserved region located in position 2658–2665 of IGF1R 3’-UTR and showed that overexpression of miR-630 reduced the mRNA and protein levels of IGF1R in the pancreatic cancer cell line, PANC-1 [16]. IGF1R mRNA was found not to be significantly altered following miR-630 manipulation; thus we propose that the predominant mechanism of action for miR-630 in breast cancer cells is likely to be at a post-transcriptional level by its inhibition of IGF1R protein translation. Following on from this observation, we subsequently investigated if post-translational modifications may be influenced by miR-630. Here we observed that inhibition of miR-630 increased IGF1R phosphorylation, while introduction of miR-630 induced its de-phosphorylation.

**Figure 6:**
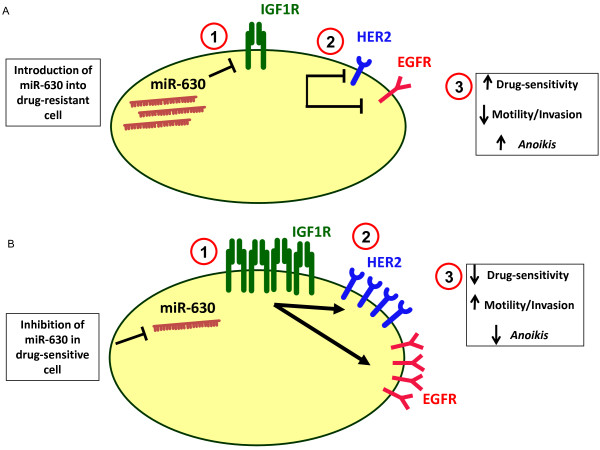
**Proposed model for manipulation of miR-630 in breast cancer cells.** Elevated IGF1R, HER2 and EGFR have been associated with drug resistance and cancer cell aggression. **(A)** Introducing miR-630 into acquired or innately resistant breast cancer cells can (1) directly inhibit the mRNA translation of IGF1R. (2) Crosstalk of IGF1R to other receptor tyrosine kinases (HER2 and EGFR) can lead to their subsequent down regulation and so (3) cause cells to become more sensitive to HER-targeting agents as well as decreasing their aggressive phenotype (in terms of motility, invasion and resistance to *anoikis*). **(B)** Conversely, inhibiting the expression of miR-630 in sensitive breast cancer cells can (1) prevent its binding to and translational suppression of target mRNA IGF1R. This causes an increase in IGF1R protein expression which in turn can (2) induce elevated HER2 and EGFR expression and as a result, (3) cells exhibit a more aggressive phenotype.

The drugs included in this study target both HER2 and EGFR. For this reason we also investigated whether miR-630 may modulate the expression of these receptors. We initially observed that the acquired lapatinib resistant cells used for this study (SKBR3-LR and HCC1954-LR) have increased levels of both HER2 and EGFR compared to their age-matched parent controls. This is supported by previous studies identifying that increased levels of HER2 or EGFR correlate with resistance to trastuzumab and other chemotherapy [32,40]. In addition to this, we observed that miR-630 inhibition induced an increase in both HER2 and EGFR, whereas over-expression of miR-630 decreased these proteins. Previous studies have indicated that crosstalk between IGF1R and other tyrosine kinases such as HER2 and EGFR can drive cancer progression and drug resistance [41-43]. In keeping with these previous studies, our findings suggest that miR-630 directly regulates IGF1R which, subsequently, leads to alterations in HER2 and EGFR potentially driving the phenotypic affects demonstrated in this study. Interestingly, we also found that, with altered phosphorylation of IGF1R following miR-630 manipulation, the phosphorylated levels of HER2 and EGFR also change. This further supports the potential of an associated cross-talk between IGF1R and these HER-family receptors following the phosphorylation of IGF1R. This suggested interaction between IGF1R and/or HER family receptors associated with miR-630 manipulation warrants further investigation. A model for our proposed mechanism for miR-630 is illustrated in Figure 6.

## Conclusions

In conclusion, our study indicates for the first time that miR-630 plays an important role in modulating response to HER-targeting drugs, as well as in overall aggressive phenotypic characteristics of HER2-overexpressing breast cancer cells. We have demonstrated that miR-630 may serve as a predictive biomarker for response to treatment in HER2-overexpressing breast cancer; with early indications from our conditioned media studies of its relevance as a minimally-invasive (as well as cell-based) biomarker. Our functional studies provide evidence that introducing miR-630 therapeutically in combination with HER-targeted drugs can help to circumvent resistance/insensitivity and reduce cancer cell aggression, thus improving overall response and so adding value to this class of drugs; through a mechanism that we unravelled as its controlling of IGF1R at a post-transcriptional level. Preclinical in vivo assessments of miR-630 as a predictive biomarker and therapeutic are now warranted.

## Abbreviations

miR-630: microRNA-630; CM: Conditioned medium; Ag: Aged parent control; LR: Lapatinib resistant; IGF1R: Insulin-like growth factor 1 receptor; HER2: Human epidermal growth factor receptor 2; EGFR: Epidermal growth factor receptor; pIGF1R: Phosphorylated IGF1R; pHER2: Phosphorylated HER2; pEGFR: Phosphorylated EGFR.

## Competing interests

JC has acted in a consultancy role for and has received honoraria and other remuneration from GSK. None of these monies were used for any of these studies and no drugs used were received as a gift from any company. All drugs used in this study were purchased. There is no other possible conflict of interest to declare.

## Authors’ contributions

CC, LOD conceived and designed the experiments. CC performed majority of the experiments SR, SB generated the acquired-resistant cell lines for study and performed some of the experiments. MG performed some of the experiments. CC, SB, SR, MG, IG, JC, LOD analysed and interpreted the data. LOD, IG, JC contributed reagents/materials/analysis tools. CC LOD wrote the paper. All authors read and approved the final manuscript.

## Supplementary Material

Additional file 1: Figure S2 IGF1R mRNA levels following miR-630 manipulation. (A) The levels of IGF1R mRNA following miR-630 inhibition were not significantly increased in (i) HCC1954-Ag or (ii) SKBR3-Ag cells compared to the negative control transfected cells. Similarly, transfection of miR-630 mimic into resistant (i) HCC1954-LR or (ii) SKBR3-LR cells did not induce a significant decrease in IGF1R mRNA compared the levels in cells transfected with the negative control mimic. Click here for file

Additional file 2: Figure S1 Densitometry for immunoblotting. Densitometry to accompany immunoblots illustrated in Figure 5. (A) Expression of (i) IGF1R (ii) HER2 and (iii) EGFR was found to be elevated in the acquired lapatinib-resistant cell lines (HCC1954-LR and SKBR3-LR) compared to the age-matched parent controls (HCC1954-Ag and SKBR3-Ag). (B) Inhibition of miR-630 induced an increase in (i) IGF1R (ii) HER2 and (iii) EGFR expression in HCC1954-Ag and SKBR3-Ag cells. (C) Introduction of miR-630 mimic induced a decrease in (i) IGF1R (ii) HER2 and (iii) EGFR in HCC1954-LR and SKBR3-LR cells. Results represent n = 3 ± SEM, where *p <0.05, **p <0.01, ***p <0.001. Click here for file
