# Supplementary figures and images for: miR-630 targets IGF1R to regulate response to HER-targeting drugs and overall cancer cell progression in HER2 over-expressing breast cancer
Source: Mol Cancer. 2014 Mar 24;13:71. doi: 10.1186/1476-4598-13-71 (PMC4234346; doi:10.1186/1476-4598-13-71)

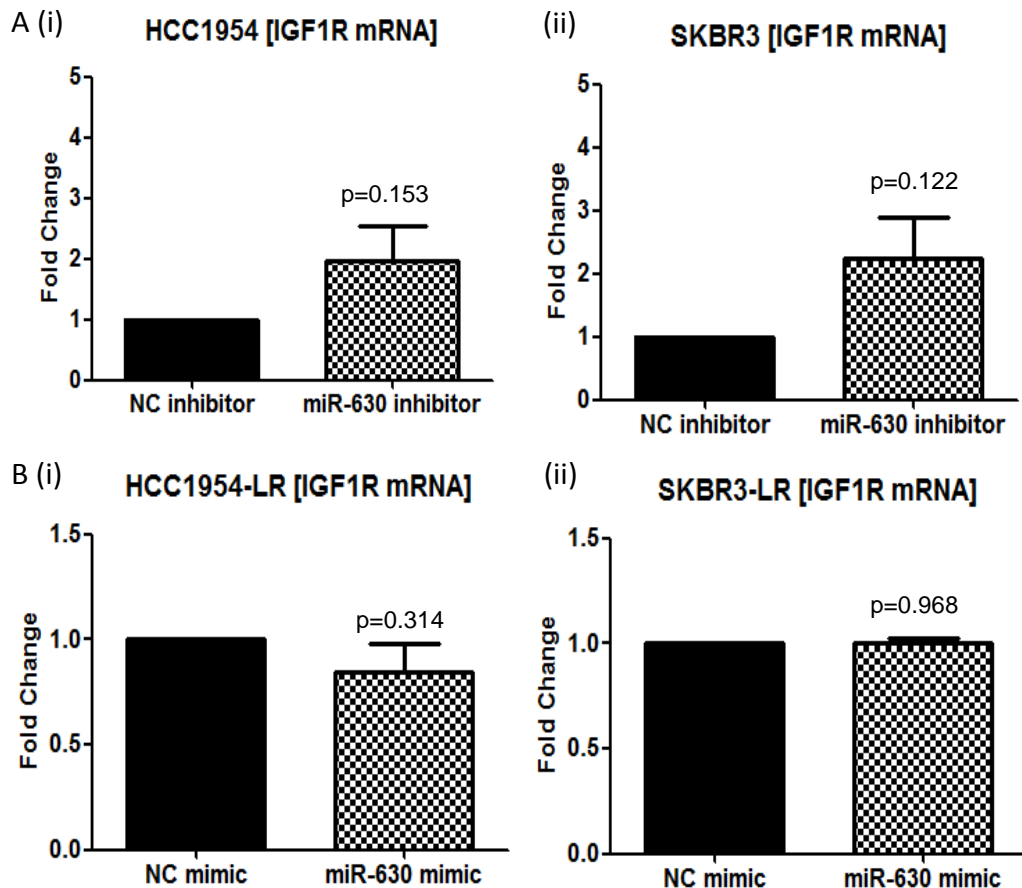

Supplement: Additional file 1: Figure S2 — IGF1R mRNA levels following miR-630 manipulation. (A) The levels of IGF1R mRNA following miR-630 inhibition were not significantly increased in (i) HCC1954-Ag or (ii) SKBR3-Ag cells compared to the negative control transfected cells. Similarly, transfection of miR-630 mimic into resistant (i) HCC1954-LR or (ii) SKBR3-LR cells did not induce a significant decrease in IGF1R mRNA compared the levels in cells transfected with the negative control mimic. [file 1476-4598-13-71-S1.pdf]

Supplementary Figure 1

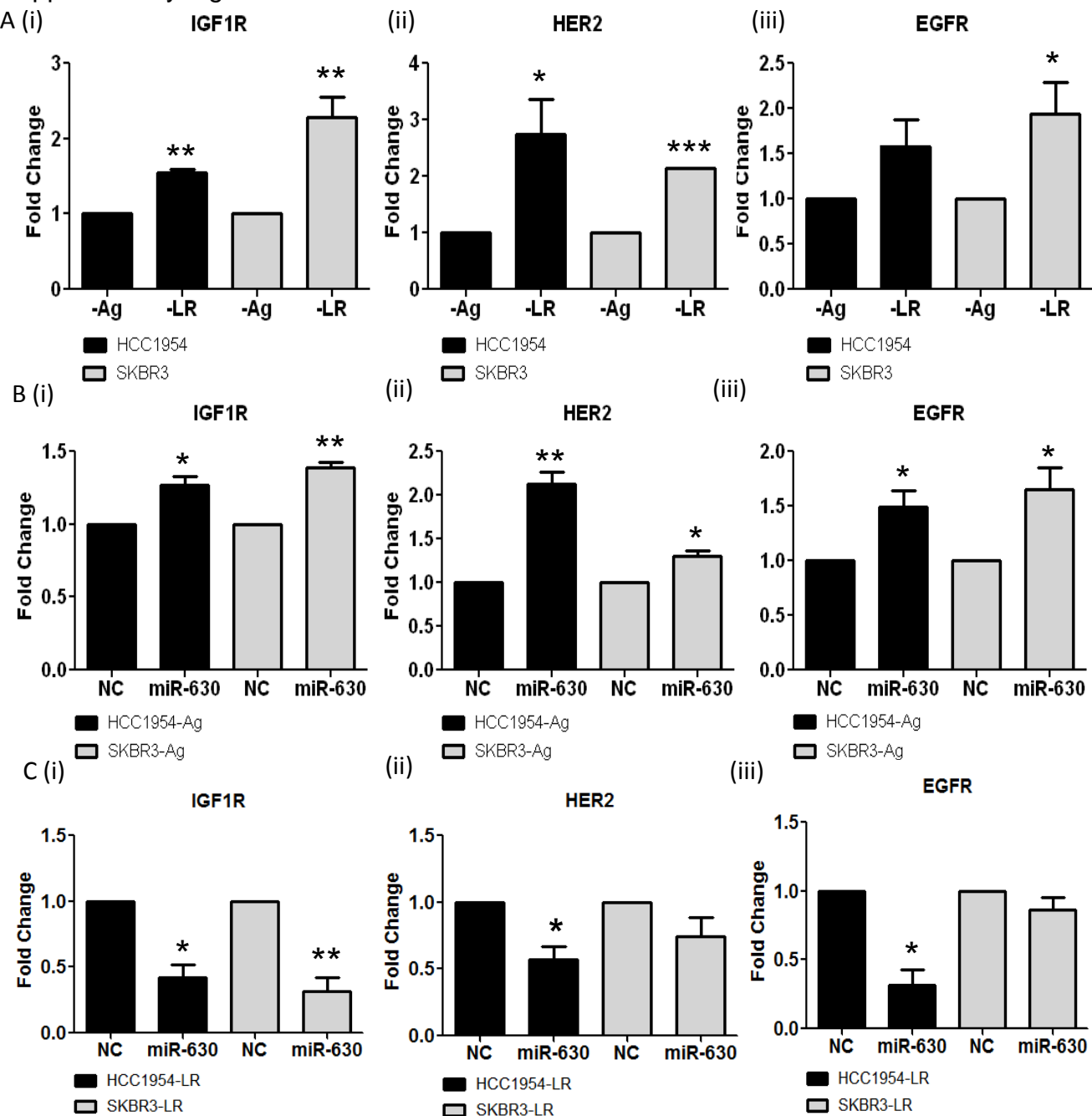

Supplement: Additional file 2: Figure S1 — Densitometry for immunoblotting. Densitometry to accompany immunoblots illustrated in Figure 5. (A) Expression of (i) IGF1R (ii) HER2 and (iii) EGFR was found to be elevated in the acquired lapatinib-resistant cell lines (HCC1954-LR and SKBR3-LR) compared to the age-matched parent controls (HCC1954-Ag and SKBR3-Ag). (B) Inhibition of miR-630 induced an increase in (i) IGF1R (ii) HER2 and (iii) EGFR expression in HCC1954-Ag and SKBR3-Ag cells. (C) Introduction of miR-630 mimic induced a decrease in (i) IGF1R (ii) HER2 and (iii) EGFR in HCC1954-LR and SKBR3-LR cells. Results represent n = 3 ± SEM, where *p <0.05, **p <0.01, ***p <0.001. [file 1476-4598-13-71-S2.pdf]
